# Genome-Wide Association Studies of Hair Whorl in Pigs

**DOI:** 10.3390/genes15101249

**Published:** 2024-09-25

**Authors:** Wenyu Jiang, Xidi Yang, Liangyu Zhu, Yiting Yang, Chengming Liu, Yong Du, Yan Wang, Lili Niu, Ye Zhao, Yihui Liu, Mailin Gan, Linyuan Shen, Li Zhu

**Affiliations:** 1 Farm Animal Genetic Resources Exploration and Innovation Key Laboratory of Sichuan Province, Sichuan Agricultural University, Chengdu 611130, China; 2022302092@stu.sicau.edu.cn (W.J.); yangxidi@stu.sicau.edu.cn (X.Y.); zhuliangyu@stu.sicau.edu.cn (L.Z.); yangyiting0914@stu.sicau.edu.cn (Y.Y.); 2022202035@stu.sicau.edu.cn (C.L.); duyong20010101@163.com (Y.D.); wangyan2023@sicau.edu.cn (Y.W.); niulili@sicau.edu.cn (L.N.); zhye@sicau.edu.cn (Y.Z.); ganmailin@sicau.edu.cn (M.G.); shenlinyuan@sicau.edu.cn (L.S.); 2 State Key Laboratory of Swine and Poultry Breeding Industry, College of Animal Science and Technology, Sichuan Agricultural University, Chengdu 611130, China; 3 Key Laboratory of Livestock and Poultry Multi-Omics, Ministry of Agriculture and Rural Affairs, College of Animal Science and Technology, Sichuan Agricultural University, Chengdu 611130, China; 4 Sichuan Province General Station of Animal Husbandry, Chengdu 610066, China; 201804594@stu.sicau.edu.cn

**Keywords:** pig, GWAS, CNV, hair whorl, hair follicle, candidate gene

## Abstract

Background: In pigs, a hair whorl refers to hairs that form a ring of growth around the direction of the hair follicle at the dorsal hip. In China, a hair whorl is considered a negative trait that affects marketing, and no studies have been conducted to demonstrate whether hair whorl affects pig performance and provide an explanation for its genetic basis. Methods: Performance-measured traits and slaughter-measured traits of hair whorl and non-hair whorl pigs were differentially analyzed, followed by genome-wide association analysis (GWAS) and copy number variation (CNV) methods to investigate the genetic basis of hair whorl in pigs. Results: Differential analysis of 2625 pigs (171 hair whorl and 2454 non-hair whorl) for performance measures showed that hair whorl and non-hair whorl pigs differed significantly (*p* < 0.05) in traits such as live births, total litter size, and healthy litter size (*p* < 0.05), while differential analysis of carcass and meat quality traits showed a significant difference only in the 45 min pH (*p* = 0.0265). GWAS identified 4 SNP loci significantly associated with the hair whorl trait, 2 of which reached genome-significant levels, and 23 candidate genes were obtained by annotation with the Ensembl database. KEGG and GO enrichment analyses showed that these genes were mainly enriched in the ErbB signaling, endothelial apoptosis regulation, and cell proliferation pathways. In addition, CNV analysis identified 652 differential genes between hair whorl and non-hair whorl pigs, which were mainly involved in the signal transduction, transcription factor activity, and nuclear and cytoplasmic-related pathways. Conclusions: The candidate genes and copy number variation differences identified in this study provide a new theoretical basis for pig breeding efforts.

## 1. Introduction

Pigs are one of the most important agricultural animals in the world and their meat traits and production performance are some of the main factors of concern for farmers and consumers [1]. In addition to this, specific traits of pigs, such as hair whorl, lameness, and umbilical hernia, are also of great concern to farmers [2,3]. In China, the hair whorl trait is regarded as a negative trait because hair whorl pigs are considered unlucky in most parts of China, largely stemming from traditional culture and folk customs. Although the pig usually symbolizes hard work and good fortune, the appearance of a hair whorl does not match people’s perceptions, with such a pig viewed as inauspicious. As a result, when pig enterprises sell breeding and commercial pigs with hair whorl features, pig vendors and farmers often refuse to buy them on their behalf, seriously affecting the trade and further weakening farmers’ incentives to breed such pigs. This not only affects the marketing of commercial pigs but also leads to the elimination of breeding pigs with hair whorls. Nearly 7% of our selected population had hair whorls, a proportion that significantly affects pig marketing. Nevertheless, there is a lack of scientific evidence to confirm whether the hair whorl trait actually affects pig performance or to provide a definitive explanation of its genetic basis.

Porcine hair whorl is due to the way skin and hair follicles form during embryonic development [4]. During the early embryonic development of the pig, skin and hair follicles begin to form on the back, accompanied by the skin curving outward to form so-called skin folds. As the embryo develops, the skin folds gradually disappear but leave behind structures that affect hair growth. When the hair follicle forms an angle with the skin surface and begins to grow upward, if the direction of hair follicle growth is different from the angle of the skin surface, a hair spiral is formed. Therefore, a hair whorl on the pig’s back is caused by the formation of skin folds and the inconsistent angle of hair follicles during embryonic development. Hair growth in pigs is an extremely complex physiological process influenced by many factors, such as the environment, nutrition, metabolic level, and gene regulation, but gene regulation is the determining factor for hair growth and development. Studies have shown that this genetic trait is controlled through polygenic inheritance, with multiple genes acting together to influence the direction of hair follicle growth [5], and some studies have shown that the growth of adipose tissue may be influenced by hair follicle growth [6]. A study of hair whorl in horses deduced that the hair whorl trait was associated with a more docile and calmer disposition [7]. These authors performed GWAS using the horse as a research model and were the first to identify a genomic region associated with the hair whorl trait in the horse in which genes were present that had neural and behavioral functions [8]. In summary, hair whorl, as an obvious genetic trait, reflects a complex embryonic developmental process, and through genetic analyses we can reveal the biological mechanisms behind it, thus contributing to a comprehensive understanding of hair whorl formation. Secondly, the polygenic genetic character of hair whorl means that, by identifying related genes, we can not only improve breeding efficiency but also develop selective breeding strategies for specific traits.

Genome-wide association studies are one of the most important tools for studying complex traits in recent years [9]. GWAS can identify genes and variant loci associated with complex traits by detecting associations between a large number of single nucleotide polymorphism (SNP) loci and phenotypes. In the field of swine research, GWAS has been widely used to study growth traits [10,11,12], reproductive traits [13,14,15], meat quality [1,16,17,18], disease resistance [19,20,21], and other traits, and a number of important results have been achieved. Copy number variation (CNV) refers to duplication and deletion variations in genomic sequences of 50 bp–5 Mb in length. In a population, the result of integration of overlapping regions of CNV among different individuals is called the CNV region (CNVR). CNV is an important source of genetic variation and can identify genetic variation among varieties [22]. CNV mainly affects gene expression and function through a variety of mechanisms. CNV is considered to be an important source of genetic variation in addition to SNPs, another important contributor to genetic variation [23]. Currently, some CNVRs affecting economically important traits in pigs have been identified. For example, Zheng et al. compared CNVs in Meishan and Duroc pigs to identify a CNVR that is only present in Meishan pigs and overlaps with reproduction-related genes [24].

The aim of this study was to investigate the genetic basis of pig hair whorl using GWAS and CNV and explore whether there are performance differences between hair whorl and non-hair whorl hogs to provide a more scientific and precise genetic improvement strategy for pig breeding.

## 2. Materials and Methods

### 2.1. Study Population and Phenotypic Determination

**Figure 1:**
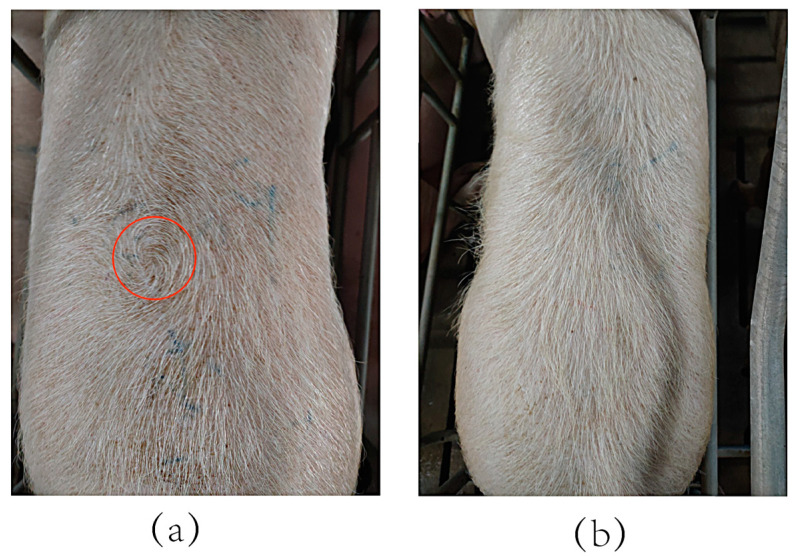
Example of hair whorl and non-hair whorl pigs: (**a**) hair whorl pig (The red circled part shows the hair whorl of the pig); (**b**) non-hair whorl pig.

The population used in this study was from a breeding farm in southwest China. A total of 2625 Canadian Duroc, Canadian Large White, and Canadian Long White pigs (667 boars and 1958 sows) born between 2020 and 2022 were used. Of these, 171 pigs were recorded as hair whorl, and 2454 pigs were recorded as non-hair whorl. In this study, the hair whorl trait was manually determined, and the criterion for the determination was to check whether the hairs on the back and rump of the pigs formed a hair whorl (Figure 1).

### 2.2. Performance Measurement and Slaughter Measurement

Performance measurement data of this population were collected, including live litter size, total litter size, healthy litter size, birth weight, left lactation size, right lactation size, corrected 100 kg day age, corrected 100 kg backfat, FCR (feed-to-meat ratio), etc. The *t*-test in GraphPad software was used to analyze whether or not there was a significant difference between inverted rotation and non-inverted rotation pigs in terms of these traits.

In this study, 6 Canadian hair whorl castrated and 6 Canadian non-hair whorl castrated Large White pig boars from a breeding farm in Southwest China were selected for slaughter performance measurement. All test pigs were around 65 kg and were weaned from food and prohibited from drinking for 24 h prior to slaughter. Pigs were slaughtered in a standard slaughtering session. After slaughter, the psoas and longissimus dorsi of the meat were taken directly from the waist position, and the meat quality characteristics were measured, respectively. Specific traits were determined, including carcass quality, meat production performance, and meat quality traits. All of the determination methods were in accordance with the technical specifications of the NY/T821-2004 pig muscle quality determination. Economic traits such as body weight and size, carcass traits, meat production performance, visceral organ rate, meat quality, and other economic traits were systematically tested, comprehensively evaluated, and comparatively analyzed in 6 hair whorl and 6 non-hair whorl castrated boars.

### 2.3. Genotyping and Quality Control

DNA was extracted from the ear tissues of 2625 pigs using a standard phenol/ chloroform method and then quantitatively diluted to 50 ng/μL. All DNA samples were genotyped using a KPS Porcine breeding chip 50 K SNP microarray from Beijing Compass Biotechnology Co., Ltd., Beijing, China, comprising a total of 57,466 SNPs, and quality control was performed using PLINKv1.90 [25] software. Quality control for SNPs involved removing those with a detection rate < 0.9, a minor allele frequency (MAF) < 0.05, and a Hardy–Weinberg equilibrium (HWE) *p*-value < 1 × 10^−6^ [26]. This resulted in the exclusion of 406 SNPs for detection rate, 4870 for MAF, and 22,305 for HWE. Autosomal SNPs (1–18) were extracted, leaving 22,267 SNPs from 2625 pigs for further analysis.

The Bonferroni correction method was used to determine the genomic significance threshold and chromosomal group significance threshold. A total of 22,267 valid SNPs were used in this study, and the genomic highly significant threshold was 0.01/N (N = the number of times the statistical test was performed, i.e., the number of SNPs); the genomic significance threshold was 0.05/N; and the chromosomal group significance threshold was 1/N. The calculated post-genomic highly significant level threshold was 4.5 × 10^−7^; the genomic significant level *p*-value was 2.25 × 10^−6^; and the chromosomal significant level threshold was 4.49 × 10^−5^.

### 2.4. Genome-Wide Association Studies

This study used a Q + K model in the GMAT software package [27] to perform GWAS analysis on 2625 pigs based on the KPS Porcine breeding chip 50 K SNP microarray. The model is expressed as follows:

(1) y=μ+Wα+xβ+Zu+e

where *y* is the vector of phenotypic values; *μ* is the population mean; *α* is the vector of fixed effects; *W* is the fixed-effects design matrix; *x* is the vector of SNPs; *β* is the SNP effect value: *e* is the randomized residual; *u* is the individual effect; *Z* is the design matrix; and $u\sim N(0,K\sigma_{a}^{2}),e\sim N(0,I\sigma_{e}^{2})$.

In this study, the Bonferroni correction method was used to adjust the significance level of the *p*-value of the GWAS results, and the “CMplot” in R software was used to plot the Manhattan plot and the Quantile–Quantile plot to represent the distribution of GWAS results [28,29].

### 2.5. Linkage Disequilibrium Analysis

We used PLINK software to perform linkage disequilibrium analysis of 5 significant SNPs found after genome-wide association analysis. The linkage disequilibrium (LD) values of the tagged SNPs with other SNPs were calculated in the range of 500 kb, where PLINK used *D* and *r*^2^ as the criteria for the linkage disequilibrium analysis. The *D* value was calculated using the following formula:

*D* = *P*(*AB*) − *P*(*A*) × *P*(*B*) (2)

where *P*(*AB*) represents the actual observed frequency of AB, and *P*(*A*) × *P*(*B*) represents the expected value of the frequency of AB.

*r*^2^ is a measure of the degree of joint genetic variation between two loci, calculated as follows:

*r*² *= D*²/(*P*(*A*)*P*(*a*)*P*(*B*)*P*(*b*)) (3)

where *D*² refers to the squared value of *D*; *P*(*A*) and *P*(*a*) represent the frequencies of the A allele and a allele at the first locus, respectively; and *P*(*B*) and *P*(*b*) represent the frequencies of the B allele and b allele at the second locus, respectively.

LDdecay maps were plotted using the Plot_OnePop.plcommand in PopLDdecay [30]. The bin1 parameter in this command indicates the distance between adjacent SNP sites, and the bin2 parameter is used to adjust the smoothness of the LD decay curve. There were 22,267 SNPS in our dataset. We set bin1 to 50 and bin2 to 1000.

LDBlockShow [31] was used to perform block mapping of significant SNPs, and 500 kb upstream and downstream of significant SNPs was selected for analysis.

### 2.6. Analysis of Copy Number Variation

The LRR-BAF values of pigs were processed into SNP number, chromosome number, SNP position, individual LRR value, and individual BAF value using Python. Copy number variation detection was performed on hair whorl and non-hair whorl pigs using PennCNV (v1.0.7) software [32], and the detected CNVs were filtered under the following conditions: the length of the CNVs was greater than 10 kb, and the smallest number of SNPs contained was three.

PennCNV uses the Hidden Markov model (HMM) with the following equation:

(4) $$P\left(r_{1},\ldots ,r_{M},b_{1},\ldots ,b_{M}\right)=\sum_{Z_{1}}\ldots \sum_{Z_{M}}\left\{\left(\prod_{i=1}^{M}P\left(r_{i}|z_{i}\right)P\left(b_{i}|z_{i}\right)\right)\left(P\left(z_{1}\right)\prod_{i=2}^{M}P\left(z_{i}|z_{i-1}\right)\right)\right\}$$

where *ri*, *bi*, and *zi* represent the logR ratio, B allele frequency, and copy number variant status at SNP *i*, respectively.

According to the copy number variants detected by PennCNV, the chromosome number, start site, and end site information of each copy number variant record were extracted, and then the coordinates of the copy number variants were merged by taking the concatenation set using the merge command of Bedtools software, and the CNVR was obtained for subsequent analysis.

### 2.7. Identification of Candidate Genes

When making the candidate gene identification, we used the R software (version 4.2.1) package “Biomart” [33] in the Ensembl database [34,35]. The candidate genes were screened for each of the significant SNPs and copy number variant segments.

### 2.8. Tional Enrichment Analysis

To further annotate functional genes near significant SNPs, we screened genes 500 kb upstream and downstream from each significant SNPs. Next, we use the DAVID annotation tool for these functional genes to carry out detailed comments [36,37,38], including Gene Ontology (GO) enrichment, Kyoto Encyclopedia of Genes and Genomes (KEGG) pathway enrichment analysis functions, and other biological database integration. In the presentation of the enrichment analysis results, we used the visualization function to present the enrichment of each pathway more clearly.

## 3. Results

### 3.1. Performance Measure

A total of 2625 pigs were recorded in this study for various performance measures. The descriptive statistics recorded are located in Table 1, and the results of the test of variance of the performance measures are presented in Table 2, which showed that there were significant differences between hair whorl and non-hair whorl pigs in the number of live piglets in the same litter, total number of piglets in the same litter, number of healthy piglets in the same litter, the corrected 100 kg body weight of age, and FCR (feed-to-meat ratio). After genome-wide association analysis of the differential traits, the detected significant SNPs did not overlap with the hair whorl-associated significant SNPs.

**Figure 2:**
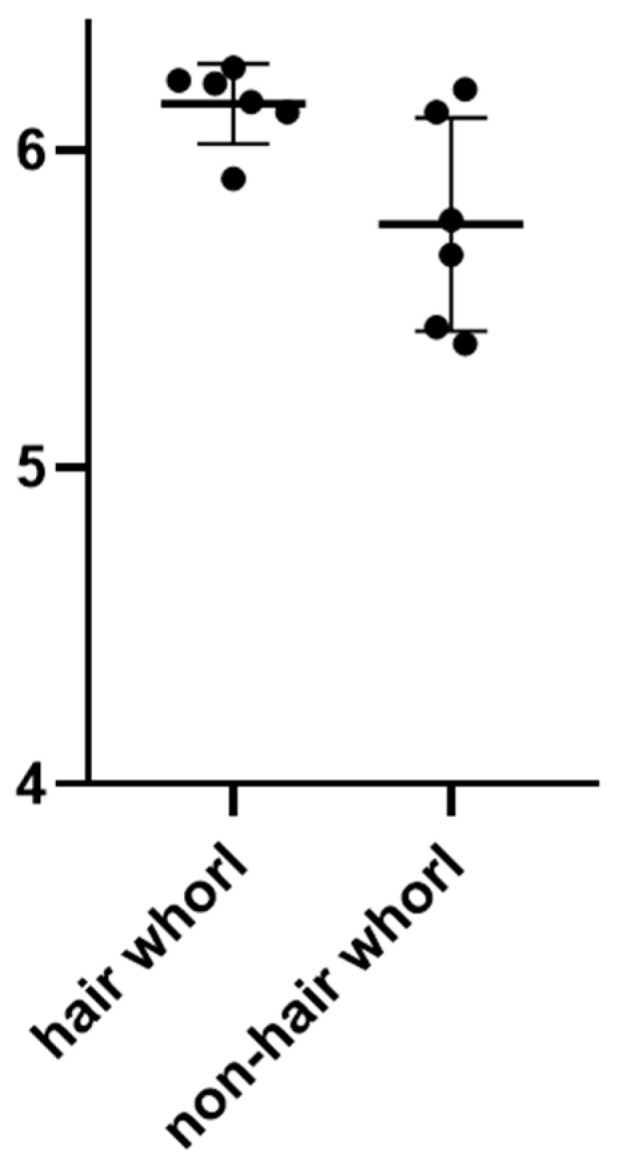
pH difference between hair whorl and non-hair whorl pigs.

The results of the slaughter measurements indicated that the 12 pigs in this assay had good overall carcass traits and meat quality traits. The slaughter rates of hair whorl and non-hair whorl pigs were relatively similar for meat production traits, but in terms of lean meat output, hair whorl pigs were slightly lower than non-hair whorl pigs. The *t*-tests using GraphPad software showed that there was no significant difference in leanness between hair whorl and non-hair whorl pigs (*p* = 0.1252); no significant difference in corrected 100 kg backfat thickness (*p* = 0.51); and no significant difference in corrected 100 kg longissimus dorsi of the meat thickness (*p* = 0.21). In terms of meat quality traits, there was a significant difference between the pH of the longissimus dorsi at 45 min after slaughter (*p* = 0.0265) (Figure 2), while the rest of the traits were not significantly different.

### 3.2. The Significant SNPs for Hair Whorl Trait

**Figure 3:**
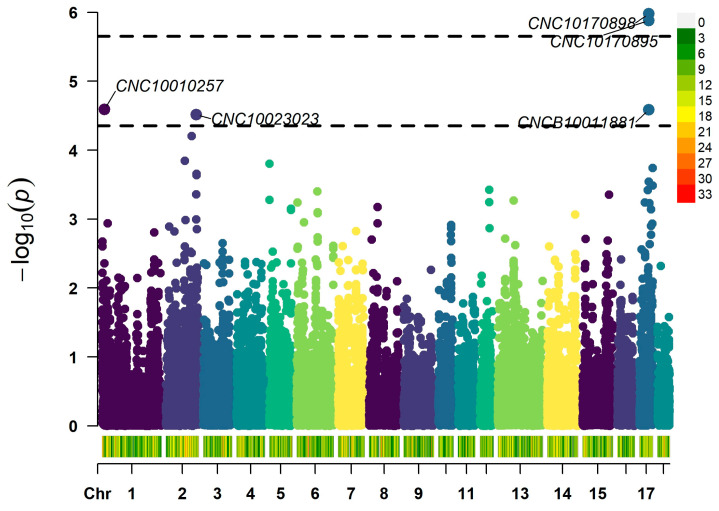
Manhattan Plot of GWAS results of the hair whorl trait.

**Figure 4:**
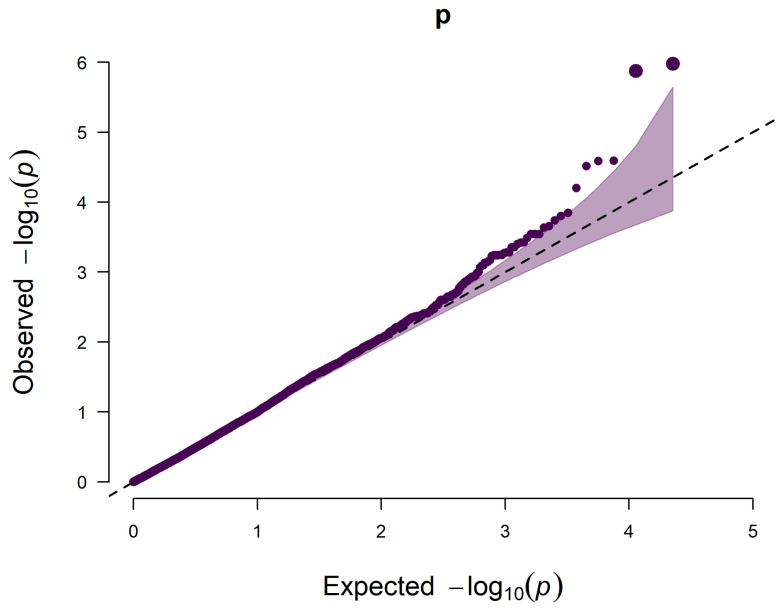
QQ plot of GWAS results for the hair whorl trait.

After SNP quality control, genome-wide association analysis was performed on 22,267 SNPs in 18 autosomal pairs. A total of 5 SNPs reaching the significance level were considered to be significantly associated with the hair whorl trait, among which CNC10170898 (chr17:44143739) and CNC10170895 (chr17:44164745) located on chromosome 17 reached the genomic significance level, and the rest that reached the genomic significance level were: CNC10010257 (chr1:9778686), CNCB10011881 (chr17:44126874), and CNC10023023 (chr2:141779308), the GWAS results are displayed Figure 3 and Figure 4.

### 3.3. Linkage Disequilibrium Results

The analysis results showed that the quality controlled SNPs in our sample complied with the Hardy–Weinberg equilibrium (HWE) law, and most SNPS had a significant LD relationship. In addition, we also found that SNP CNC10170898 and CNC10170895 had a highly tight LD relationship (r^2^ = 0976), and these 2 SNPS may contribute significantly to the hair whorl trait.

**Figure 5:**
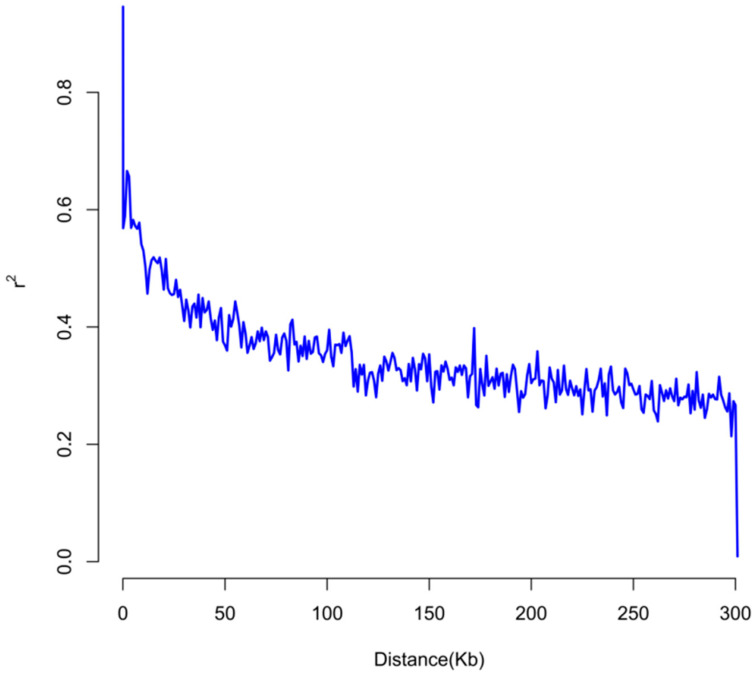
LD attenuation diagram.

The LD attenuation map showed that the LD values between the sites decreased with increasing distance between them, and the image showed a relatively smooth trend (Figure 5). This indicated that the LD relationship between these sites was relatively weak in the study sample, and there was no dramatic LD change point. This relatively smooth trend usually enabled us to determine the LD situation among SNP sites more accurately, which provided valuable data support for the study of the genome structure of the samples and further association analysis.

**Figure 6:**
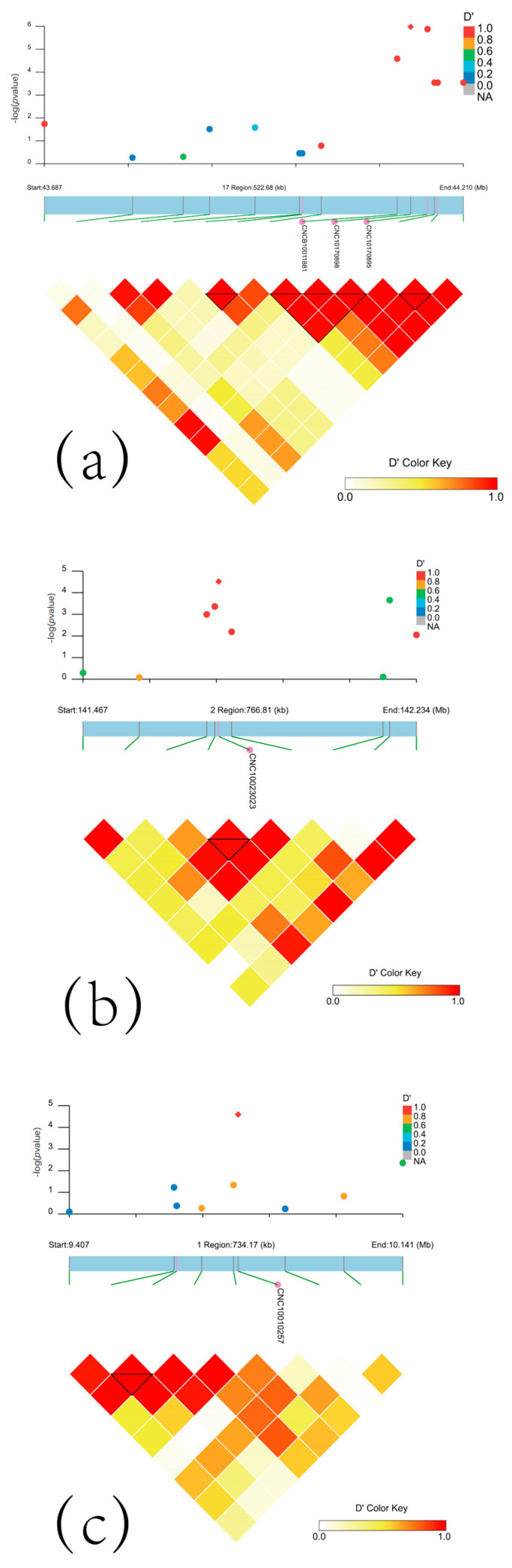
Significant SNP linkages: (**a**) chr17 43. 68 mb−44.21 mb haplotype block diagram; (**b**) chr2 141.467 MB−142.234 mb haplotype block diagram; (**c**) chr1 9.47 Mb−10.141 mb haplotype block diagram. The square shaped points in the figure are the most significant snp loci of this GWAS.

We used the LDBlockShow package to map 500 kb upstream and downstream of salient sites on chromosomes 1, 2, and 17 (Figure 6). The results showed that the two salient sites on chromosome 17 formed blocks, and the significant SNP sites on chromosome 2 also formed blocks. However, the significant sites located on chromosome 1 failed to form blocks, which may be due to the presence of false positives at this site. Therefore, we deleted this SNP loci.

### 3.4. Results of Copy Number Variation Analysis

Copy number variation detection of hair whorl and non-hair whorl pigs using PennCNV showed that 1527 CNV records were detected in hair whorl pigs, of which 1279 were CNVs of the loss type and 248 were CNVs of the gain type. A total of 20,249 CNV records were detected in non-hair whorl pigs, with 17,787 CNVs of the loss type and 2462 CNVs of the gain type (Table 3).

### 3.5. Candidate Gene and Functional Annotation

#### 3.5.1. GWAS Results

Candidate gene screening for significant SNPs was performed using the pig gene sequence in the Ensembl database with the “Biomart” package in R (version 4.2.1) software for each significant SNP. CNC10170898 (chr17: 44143739), CNC10170895 (chr17: 44164745), and CNCB10011881 (chr17:44126874) all fell on CHD6 on chr17; CNC10023023 (chr2:141779308) fell on NRG2 on chr2 (Table 4).

Subsequently, 500 kb upstream and downstream of 4 significant SNPs were identified as candidate genes, and 23 candidate genes were obtained, and pathway enrichment analysis of the above candidate genes was carried out using DAVID (Table 5). The KEGG pathway enrichment results showed that there were three pathways, and the GO pathway enrichment results showed that there were eight pathways, of which there were five pathways with *p* < 0.05.

SNP molecular markers significantly associated with the pig hair whorl trait were screened by genome-wide association analysis, 4 SNP loci significantly associated with the pig hair whorl trait were screened, the number of individuals of different genotypes and the number of corresponding genotypes of hair whorl were further counted at each SNP locus, and the hair whorl rate of different genotypes (number of individuals with hair whorl trait/number of individuals of the genotype) was calculated. As shown in Table 6, for the four SNP loci 17:CNC10170898, 17:CNC10170895, 17:CNCB10011881, and 2:CNC10023023, the frequency of pigs with the hair whorl trait was higher than that of individuals with other genotypes when the genotypes were AA, CC, TT, and GG, respectively (Table 6).

#### 3.5.2. CNV Results

Candidate gene screening for copy number variation in hair whorl and non-hair whorl pigs was performed using pig gene sequences in the Ensembl database with the “Biomart” package in R (version 4.2.1) software. There were 582 candidate genes for hair whorl pigs and 1228 candidate genes for non-hair whorl pigs. The screening resulted in 652 differential genes. 

**Figure 7:**
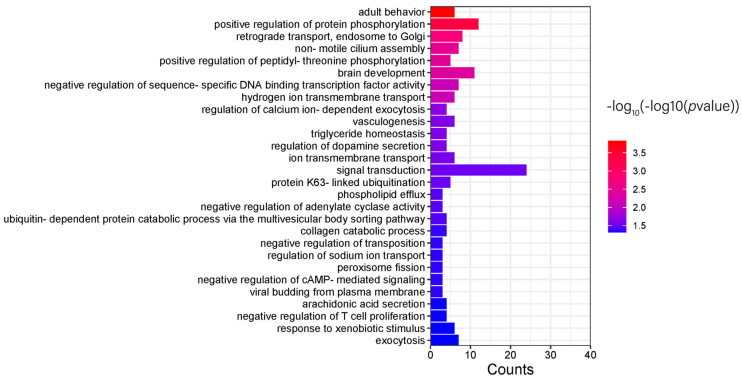
GO−BP pathway enrichment results.

**Figure 8:**
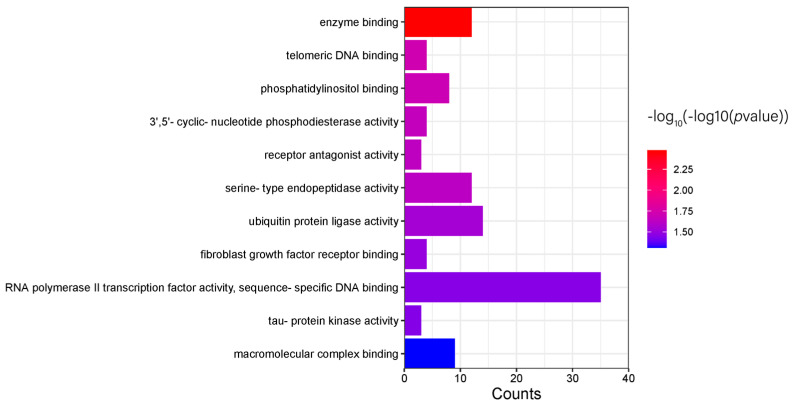
GO−MF pathway enrichment results.

**Figure 9:**
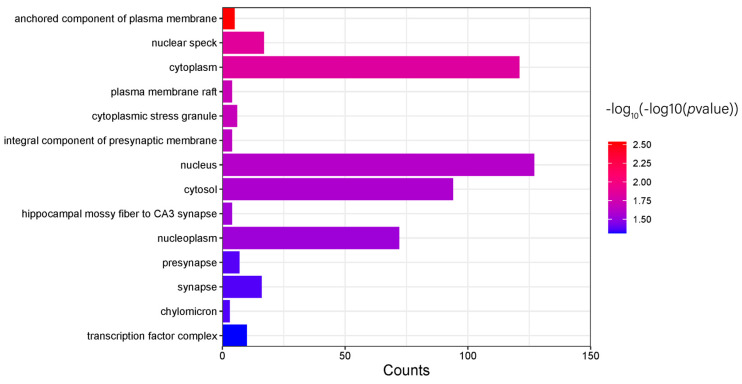
GO−CC pathway enrichment results.

The differential genes obtained were analyzed by pathway enrichment using DAVID. The KEGG pathway enrichment results showed 8 pathways, but the number of pathways with *p* < 0.05 was 0. The GO pathway enrichment results showed 92 pathways, of which 53 pathways had *p* < 0.05, including 28 that belonged to Biological Process (BP) pathways (Figure 7), 11 that belonged to Molecular Function (MF) pathways (Figure 8), and 14 that belonged to Cellular Component (CC) pathways (Figure 9). The results of the enrichment analysis were visualized.

## 4. Discussion

A number of localization factors have led to the unpopularity of hair whorl pigs for selection and retention; however, no studies have shown that hair whorl pigs perform poorly in terms of production performance. In our study, the total litter size, live litter size, healthy litter size, corrected 100 kg day of age, and corrected FCR were significantly different (*p* < 0.05) between hair whorl and non-hair whorl pigs, and our genome-wide association analyses of these differing performance measurement traits revealed that the significant SNPs detected did not overlap with the hair whorl trait-related significant SNPs. This suggests that the hair whorl trait may be related to different mechanisms of inheritance than these traits, independent of each other, or the complexity of the samples and phenotypes resulted in insignificant genetic associations. It may also be related to biases in statistical analysis methods or sample selection. This may require further exploration of potential genetic links between traits in conjunction with functional genomics. In this study, we identified 4 SNP loci significantly associated with the hair whorl trait, and a total of 23 candidate genes were identified within 500 kb upstream and downstream of them, with a total of eight pathways enriched (*p* < 0.05).

Positive regulation of the endothelial apoptotic process (GO:2000353) refers to the enhancement or promotion of programmed endothelial cell death through various biochemical and molecular mechanisms. Several studies have shown that apoptosis plays an important role in the regulation of hair follicle and vascular degeneration [39]. The ErbB signaling pathway (KEGG:ssc04012) refers to multiple processes that dimerize or heterodimerize members of ErbB through binding to a variety of signaling molecules and promote autophosphorylation and downstream signaling cascades. Epidermal growth factor receptor (GO:0007173) is a prototypical member of the ErbB family of receptor tyrosine kinases, activated by ligand-dependent homo- or heterodimerization [40]. Histological studies have shown that EGFR is widely distributed in epidermal derivatives of the epidermis and skin appendages and is particularly restricted to the ORS of sebaceous glands and anagen hair bulbs [41,42]. Many studies have shown that mutant or transgenic mice associated with EGFR or its ligands have altered hair follicle development [43], and Klufa et al. have shown that mice with constitutive deletion of EGFR in the epidermis develop severe cutaneous inflammation, and that the hair follicle is the epidermal structure where the inflammation begins [44]. Glutamatergic synapses (GO:0098978) are synapses that use glutamate (Glu) as the primary neurotransmitter. Robert et al. identified a glutamatergic regulatory system with the presence of synaptic-like vesicles in the lanceolate terminals of the hair follicles in mice and rats [45].

The results of the copy number variation analysis and GO pathway enrichment of differential candidate genes in hair whorl versus non-hair whorl pigs showed the enrichment of genes related to signal transduction, transcription factor activity, the nucleus, and cytoplasm in copy number variation. These results provide clues for a deeper understanding of the mechanisms by which copy number variation affects porcine the hair whorl trait. Further studies could focus on exploring the specific functions of these enriched pathways and subcellular localization-related genes and their relationships with the porcine hair whorl trait, thus deepening our understanding of the regulation of this trait.

## 5. Conclusions

In this study, genome-wide association analysis and copy number variation analysis were performed on the basis of performance measurements, which showed significant differences between hair whorl pigs and non-hair whorl pigs in the number of live piglets in the same litter, total number of piglets in the same litter, number of healthy piglets in the same litter, corrected 100 kg body weight of age, and FCR (feed-to-meat ratio). On this basis, slaughter measurements were carried out to test whether carcass and meat quality differences existed between hair whorl and non-hair whorl pigs, and the results of the analysis of variance after the slaughter results showed that hair whorl and non-hair whorl pigs performed well in terms of carcass and meat quality, and only slight differences existed. After screening, genome-wide association analysis detected a total of 4 significant SNPs on SSC2 and SSC17. For these 4 SNP loci, with genotypes AA, CC, TT, and GG, the frequency of pigs with the hair whorl trait was higher than that of individuals without the hair whorl trait.

Based on the identification of upstream and downstream 500 kb candidate genes, 23 possible genes were screened as candidate genes for the hair whorl trait in pigs, and 8 significant signaling pathways were enriched (*p* < 0.05), which provided a genetic basis for the subsequent enhancement of breeding through genomic breeding technology.

Copy number variation results identified 652 differential genes between hair whorl and non-hair whorl pigs, which were mainly enriched in signal transduction, transcription factor activity, and nuclear and cytoplasmic-related pathways.

## Figures and Tables

**Table 1:** Descriptive statistics of performance measurement data.

Trait	Individuals	Min	Mean	Max	SD
Number of Live litter size	2096	1	12.27	21	3.57
Number of Total litter size	2097	1	12.88	21	3.74
Number of healthy litters	2096	1	10.93	18	3.09
Birth weight	2067	0.6	1.25	2.2	0.25
Number of left teats	2096	5	7.04	9	0.61
Number of right teats	2096	5	6.91	9	0.58
Corrected 100 kg weight of age	1851	129.89	159.79	232.79	18.33
Corrected 100 kg backfat	1851	5.11	12.15	24.39	2.81
Corrected FCR	659	1.41	1.85	3.17	0.30

**Table 2:** Analysis of performance measurement variability results.

Trait	Num of Hair Whorl Pigs	Mean	Num of Non-Hair Whorl Pigs	Mean	*p* Value
Number of Live litter size	128	13.23	1968	12.21	0.0018
Number of Total litter size	128	13.94	1969	12.81	0.0009
Number of healthy litters	128	11.69	1968	10.88	0.0043
Corrected 100 kg weight of age	128	164.49	1723	159.44	0.0026
Corrected FCR	53	1.73	606	1.86	0.0015
Birth weight	128	1.22	1939	1.25	0.1429
Number of left teats	128	7.02	1968	7.05	0.6032
Number of right teats	128	6.90	1968	6.91	0.7724
Corrected 100 kg backfat	128	11.99	1723	12.16	0.5146

**Table 3:** Results of copy number variation detection of PennCNV.

Type	Hair Whorl	Non-Hair Whorl
Losses	1279 (83.76%)	17,787 (87.84%)
Gains	248 (16.24%)	2462 (12.16%)
Total	1527	20,249

**Table 4:** Candidate genes corresponding to significant SNPs.

SNP	SSC	Position	*p* Value	Alleles	Candidate Gene	Start	End
CNC10170898	17	44143739	1.05 × 10^−6^	G/A	CHD6	44048098	44265431
CNC10170895	17	44164745	1.32 × 10^−6^	T/C	CHD6	44048098	44265431
CNCB10011881	17	44126874	2.60 × 10^−5^	C/T	CHD6	44048098	44265431
CNC10023023	2	141779308	3.05 × 10^−5^	T/G	NRG2	141662369	141824727

**Table 5:** Significant gene ontology (GO) terms and Kyoto Encyclopedia of Genes and Genomes (KEGG) pathways associated with the hair whorl trait in pigs.

Term	Count	*p*-Value	Genes
GO:2000353 positive regulation of endothelial cell apoptotic process	2	0.011625061	*ECSCR*, *PLCG1*
GO:0005654 nucleoplasm	7	0.022952206	*PROB1*, *ZHX3*, *STING1*, *SPATA24*, *CHD6*, *ECSCR*, *CXXC5*
GO:0008284 positive regulation of cell population proliferation	3	0.023643003	*PURA*, *MZB1*, *HBEGF*
GO:0098978 glutamatergic synapse	3	0.031915019	*PURA*, *PSD2*, *PLCG1*
GO:0007173 epidermal growth factor receptor signaling pathway	2	0.034496111	*PLCG1*, *HBEGF*
GO:0032587 ruffle membrane	2	0.063070664	*PSD2*, *PLCG1*
GO:0140297 DNA-binding transcription factor binding	2	0.075270787	*PURA*, *CXXC5*
GO:0098794 postsynapse	2	0.077077652	*PURA*, *PSD2*
ssc04012 ErbB signaling pathway	3	0.001177119	*NRG2*, *PLCG1*, *HBEGF*
ssc05171 Coronavirus disease—COVID-19	3	0.010096157	*STING1*, *PLCG1*, *HBEGF*
ssc01521 EGFR tyrosine kinase inhibitor resistance	2	0.04920922	*NRG2*, *PLCG1*

**Table 6:** Genotype frequency and hair whorl rate distribution of candidate SNP loci in pigs.

SNP	Number	Genotype Frequency (Hair Whorl Rate)		
		0/0	0/1	1/1
Chr17:CNC10170898	2625	GG:0.8690 (0.04910)	AG:0.1265 (0.1626)	AA:0.004571 (0.4167)
Chr17:CNC10170895	2625	TT:0.8659 (0.04927)	CT:0.1295 (0.1588)	CC:0.004571 (0.4167)
Chr17:CNCB10011881	2625	CC:0.8270 (0.05157)	TC:0.1665 (0.1236)	TT:0.006095 (0.3125)
Chr2:CNC10023023	2625	TT:0.8827 (0.04704)	GT:0.1070 (0.1922)	GG:0.01029 (0.2963)

## Data Availability

The original contributions presented in the study are included in the article, further inquiries can be directed to the corresponding author.
